# The Effects of Goal Relevance and Perceptual Features on Emotional Items and Associative Memory

**DOI:** 10.3389/fpsyg.2017.01223

**Published:** 2017-07-25

**Authors:** Wei B. Mao, Shu An, Xiao F. Yang

**Affiliations:** Emotion and Cognition, School of Psychology, Shandong Normal University Jinan, China

**Keywords:** emotion, goal relevance, item salience, item familiarity, associative memory

## Abstract

Showing an emotional item in a neutral background scene often leads to enhanced memory for the emotional item and impaired associative memory for background details. Meanwhile, both top–down goal relevance and bottom–up perceptual features played important roles in memory binding. We conducted two experiments and aimed to further examine the effects of goal relevance and perceptual features on emotional items and associative memory. By manipulating goal relevance (asking participants to categorize only each item image as living or non-living or to categorize each whole composite picture consisted of item image and background scene as natural scene or manufactured scene) and perceptual features (controlling visual contrast and visual familiarity) in two experiments, we found that both high goal relevance and salient perceptual features (high salience of items vs. high familiarity of items) could promote emotional item memory, but they had different effects on associative memory for emotional items and neutral backgrounds. Specifically, high goal relevance and high perceptual-salience of items could jointly impair the associative memory for emotional items and neutral backgrounds, while the effect of item familiarity on associative memory for emotional items would be modulated by goal relevance. High familiarity of items could increase associative memory for negative items and neutral backgrounds only in the low goal relevance condition. These findings suggest the effect of emotion on associative memory is not only related to attentional capture elicited by emotion, but also can be affected by goal relevance and perceptual features of stimulus.

## Introduction

Although, researches on individual separate item memory generally proposed that emotional events are more likely to be remembered than neutral ones (Raymark et al., 2001; Sharot et al., 2004; Mather and Knight, 2005; LaBar and Cabeza, 2006; Luminet and Curci, 2009; Zebrowitz et al., 2015; Mammarella et al., 2016a), prior studies on associative memory drew inconsistent conclusions (Doerksen and Shimamura, 2001; Madan et al., 2012; Bisby and Burgess, 2014). Some studies reported that emotion could enhance associative memory. For instance, people were better at remembering the color or location of emotional words than that of neutral words (Doerksen and Shimamura, 2001; Kensinger and Corkin, 2003; D’Argembeau and Van der Linden, 2005; Mather et al., 2009; Schmidt et al., 2011; Mammarella et al., 2016b). In contrast, other studies suggested that emotion could impair associations between emotional item and other disparate item or background scene information (Touryan et al., 2007; Mather and Knight, 2008; Guillet and Arndt, 2009; Madan et al., 2012; Bisby and Burgess, 2014). According to attentional narrowing hypothesis (Easterbrook, 1959), Mather (2007) further proposed emotion may narrow attention and selectively draw attention to the arousing aspects of an event, then such focused attention to emotional items will enhance the binding between the item and its intrinsic properties, while impair the binding between items and items or between items and backgrounds.

Though the attentional narrowing account above mentioned seems reasonable, it still can not account for all findings. Mather et al. (2009) found that when two pictures were paired presented together, arousing pictures did not affect item and location memory for the other non-arousing pictures in their Experiment 1 and Experiment 2. In their Experiment 3, they further manipulated perceptual dominance of one of two pictures and presented less interesting and less prominent background patterns paired with pictures, then they found that arousing pictures would impair memory for less prominent background patterns paired with themselves. Obviously, this result suggested that arousing central items in scenes are more likely to impair memory for peripheral background information (Christianson et al., 1991; Hornstein et al., 2003). Similarly, Kensinger et al. (2007) also found that participants usually showed memory enhancement in central negative items but memory impairment in surrounding backgrounds. Together, this pattern of findings seems to suggest that an emotional item may not affect memory for information that is equally perceptually salient prominent but is likely to impair memory for information that is less perceptually prominent than itself. Therefore, besides the arousal of items, perceptual features associated with arousing items seem to play a key role.

Indeed, except of objective perceptual features of stimulus, such as the location, color and size of objects, familiarity is also can be regarded as a kind of perceptual features acquired by relearning or re-exposing stimulus (Begg et al., 1992; Westerman et al., 2002). Biedenkapp and Rudy (2007) showed that rats could quickly establish successful fear conditioning with shock in familiar contexts rather than new contexts. Funk and Hupbach (2014) further found that familiarity of backgrounds could promote the binding between emotional items and background scenes in memory. However, could familiarity of item images thus enhance the binding of emotional item images and background scenes? Up till now, no previous study has investigated this question.

Additionally, Sakaki et al. (2013) suggested that stimuli relevant to current goals would gain a memory advantage over irrelevant stimuli in the emotional condition. In this study, participants were required to learn object image sequences that included several neutral objects and one perceptual oddball object (either emotional or neutral). In the high goal relevance condition, participants were told to focus on the oddball-1 objects, whereas in the low goal relevance condition, participants were told to focus on oddball or oddball+1 objects. Overall, the results demonstrated that emotion induced by oddball objects facilitated memory for the oddball-1 objects in the high goal relevance condition, but impaired memory for oddball-1 objects in the low goal relevance condition. Similarly, results from Montagrin et al. (2013) also supported that emotion could strengthen the memory for high goal relevance items, but weaken the memory for low goal relevance items.

Especially, many researches also have ever explored the roles of both goal relevance and perceptual features in visual search tasks. According to the biased competition model (Desimone and Duncan, 1995), selectivity in visual search can be influenced by two factors: one is bottom–up biases that are based on perceptual features of stimulus, the other is top–down biases that are determined by the individual’s goals. Schreij et al. (2008) found that goal-driven factors could override stimulus-driven factors, allowing people to select only the goal-driven visual items without being distracted by irrelevant conspicuous objects. However, Folk et al. (2002) proposed that the two factors interacted with each other. They found although salient perceptual features of stimulus could attract attention involuntarily, such attentional capture still would be modulated by top–down attentional control. Obviously, such outcomes suggested that the effect of perceptual features on attention might be modulated by goal relevance (Lu and Han, 2009; Mcmains and Kastner, 2011; Melloni et al., 2012). Given that attention in encoding plays important roles in the memory formation, it is reasonable to predict an interaction of goal relevance and perceptual features in their effects on associative memory.

Therefore, we wanted to further explore how both goal relevance and perceptual features would influence associative memory between emotional central item and peripheral background, especially how goal relevance would work when perceptual features were manipulated by visual salience (from physical contrast) and visual familiarity (from prior experience). According to Kensinger et al. (2007) and Funk and Hupbach (2014), the present study used complex composite pictures composed of a negative or a neutral item image superimposed onto a neutral background scene as materials in two experiments. In Experiment 1, we manipulated perceptual feature of item images by controlling their size and spatial location in backgrounds, and manipulated goal relevance of item images by controlling participant’s attention to item images or the whole composite pictures consisted of item images and backgrounds. In Experiment 2, we manipulated perceptual feature of item images by controlling their familiarity through repeating stimulus, and manipulated goal relevance of item images in the same way as Experiment 1. Our prediction was that high goal relevance might enhance emotional item memory but impair associative memory of emotional items and neutral backgrounds. Meanwhile, the effect of perceptual features on associative memory for emotional item might be modulated by goal relevance. Further, we speculated that both item salience and item familiarity might enhance emotional item memory, whereas they influenced associative memory of emotional items and neutral backgrounds in a different way.

## Experiment 1

### Method

#### Participants

Thirty two healthy undergraduate students (7 males and 25 females), aged 17–22 (*M* = 18.78, *SD* = 0.83), were recruited from Shandong Normal University, with normal or corrected-to-normal vision. All participants gave signed consent (approved by the local ethic committee) and received a gift after experiment.

#### Materials

Stimuli in this experiment were colorful complex visual pictures that were created by placing negative and neutral items onto neutral background scenes. All items and background scenes were selected from International Affective Picture System and Chinese Affective Picture System (IAPS; Lang et al., 1999; CAPS; Bai et al., 2005). Negative and neutral item images were chosen on the basis that they depicted a central object or person with few contextual details. All neutral background scenes were chosen on the basis that they did not include any people or obvious objects. All pictures were controlled for size (item: 200 × 200 pixel size; background: 735 × 500 pixel size). To make sure that valence, arousal, familiarity, visual vividness, complexity dimensions for every item image and background scene were controlled, we firstly conducted a pilot study using a 15-undergraduate group to rate every item image and background scene respectively from 1 (extremely unpleasant) to 9 (extremely pleasant) for valence, from 1 (extremely calm) to 9 (highly arousing) for arousal, from 1 (unfamiliar) to 4 (pretty familiar) for familiarity, from 1 (very not vivid) to 4 (very vivid) for visual vividness, and from 1 (pretty simple) to 4 (pretty complex) for complexity. Secondly, according to the normative data gathered for the IAPS, we picked up 112 negative item images (mean valence = 2.16 ± 1.23, mean arousal = 6.86 ± 1.65), 112 neutral item images (mean = 4.98 ± 0.54, mean arousal = 2.94 ± 1.59), and 168 neutral background scenes (mean valence = 5.07 ± 0.92, mean arousal = 3.80 ± 1.85). Lastly, we created 112 item-background composite pictures by superimposing negative or neutral item images onto neutral background scenes and asked 15 graduate students to rate the correlation of these images on a seven-point scale (from unrelated to high related). The composite pictures were low related between items and backgrounds and balanced on familiarity (*M* = 2.81, *SD* = 0.93), visual vividness (*M* = 3.22, *SD* = 0.78), and complexity (*M* = 2.12, *SD* = 0.89).

One hundred and twelve item-background composite pictures were used as study materials, including 56 negatively arousing (mean valence = 2.10 ± 1.18, mean arousal = 6.62 ± 1.67) and 56 neutral non-arousing (mean valence = 4.93 ± 1.07, mean arousal = 3.50 ± 1.91) composite pictures. In addition, we manipulated perceptual-salience of items images as high salience composite pictures and low salience composite pictures by controlling size and spatial location of item images in backgrounds. We used saliency map software (Itti and Koch, 2000) to check for salience and found that the item image which was bigger and located spatially central on neutral background scene was more salient in each composite picture. For each participant, half of composite pictures consisted of bigger size item images (200 × 200 pixel size) spatially central on neutral background scenes and the other half consisted of smaller size items (150 × 150 pixel size) spatially peripheral on neutral background scenes. The stimuli were counterbalanced across participants for each item-background composite picture consisted of same item image in the high salience condition and low salience condition.

Test materials consisted of 112 item images (56 studied item images and 56 new item images) and 168 background scenes (56 studied intact background scenes presented with these 56 item images in study phase, 56 studied rearranged background scenes presented with the other 56 item images in study phase and 56 new ones that did not appear in study phase). The item images of each category included 28 negative and 28 neutral item images, and there were 14 negative high salience item images, 14 negative low salience item images, 14 neutral high salience item images and 14 neutral low salience item images respectively.

#### Procedure

**FIGURE 1:**
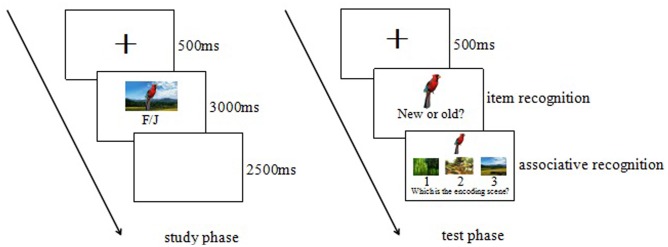
The procedure for study phase and test phase.

In the study phase, participants were told that they would view a series of pictures on computer screen and make corresponding choice for each picture. After a central fixation cross was presented for 500 ms, 112 item-background composite pictures were presented for 3000 ms one by one. We manipulated goal relevance of item images by controlling participant’s attention just to item images or to the whole composite pictures as high relevance and low relevance in two blocks. In the high goal relevance blocks, participants were asked to categorize each item image as living or non-living by pressing F key or J key; while in the low goal relevance blocks, they were just asked to categorize each whole composite picture consisted of an item image and a background scene as natural scene or manufactured scene by pressing F key or J key. There were 2 min for participants to rest between 2 blocks and the study order of 2 blocks was counterbalanced across participants. In each block, participants were presented with 28 negative composite pictures and 28 neutral composite pictures. Half of them consisted of high salience item images and the other half consisted of low salience item images (see **Figure 1**).

After the study phase, participants were exposed to a 5-min task of completing 80 math questions as distractors.

During test phase, participants were firstly required to take an old–new recognition when 56 studied item images mixed with 56 new item images were presented randomly. Following the participant’s response, we used a cued association memory test for each studied item which was recognized as old item to test the memory for the background originally intact-presented with the item by a three alternative forced choice recognition task, one was the background intact-presented with the item, another was a background from study phase that had been presented with a different item image, and the third was a novel background not seen in the study phase. Participants were instructed to select the background that had been paired with the item image in the study phase (see **Figure 1**).

### Results and Discussion

#### Item Memory

**FIGURE 2:**
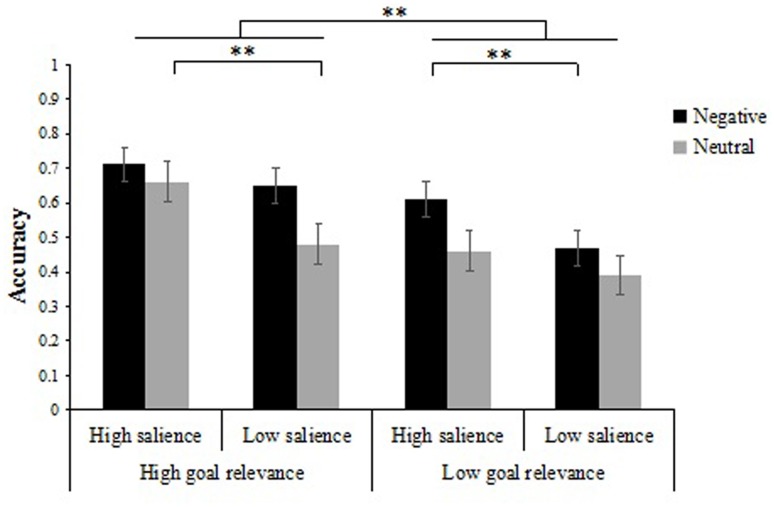
Recognition accuracy for items as a function of goal relevance, item salience, and emotion type. Error bars represent standard error. ^∗∗^*p* < 0.01.

We analyzed the proportion of correct responses on the item memory test (**Table 1**) using a 2 × 2 × 2 repeated measures analysis of variance (ANOVA) with emotion type (negative vs. neutral), item salience (low salience vs. high salience) and goal relevance (low relevance vs. high relevance) as within-participant factors. Results showed a significant emotion type × item salience × goal relevance interaction [*F*(1,31) = 7.74, *p* < 0.01, $\eta_{p}^{2}$ = 0.20]. To further analyze the interaction, we performed separate 2 × 2 ANOVAs on recognition of the items. Analysis of negative item memory performance was performed with item salience (low salience vs. high salience) and goal relevance (low relevance vs. high relevance) entered as within-participants factors. The results revealed a main effect of item salience [*F*(1,31) = 16.52, *p* < 0.01, $\eta_{p}^{2}$ = 0.35], a main effect of goal relevance [*F*(1,31) = 24.50, *p* < 0.01, $\eta_{p}^{2}$ = 0.44], and a two-way interaction that was marginally significant [*F*(1,31) = 4.00, *p* = 0.054, $\eta_{p}^{2}$ = 0.11]. Further simple-effects analyses showed that, when items were negative, memory performance for high goal relevance items was always higher than that of low goal relevance items no matter in the high salience condition or in the low salience condition [*F*(1,31) = 9.00, *p* < 0.01, $\eta_{p}^{2}$ = 0.23; *F*(1,31) = 31.00, *p* < 0.01, $\eta_{p}^{2}$ = 0.50]. Likewise, we also analyzed the effect of item salience and found in the low goal relevance condition, memory performance was greater for negative high salience items compared to negative low salience items [*F*(1,31) = 29.18, *p* < 0.01, $\eta_{p}^{2}$ = 0.49], while memory performance did not differ between negative high salience items and negative low salience items in the high goal relevance condition [*F*(1,31) = 3.28, *p* > 0.05] (**Figure 2**).

**Table 1:** Accuracy for item memory as a function of goal relevance, item salience, and emotion type (*M* ± *SD*).

	High goal relevance		Law goal relevance	
		
	High	Low	High	Low
	salience	salience	salience	salience
Negative	0.71 ± 0.17	0.65 ± 0.15	0.61 ± 0.11	0.47 ± 0.13
Neutral	0.66 ± 0.17	0.48 ± 0.18	0.46 ± 0.11	0.39 ± 0.17


Results from participants’ neutral item memory performance using a similar 2 × 2 ANOVA of item salience (low salience vs. high salience) and goal relevance (low relevance vs. high relevance) showed a main effect of item salience [*F*(1,31) = 16.61, *p* < 0.01, $\eta_{p}^{2}$ = 0.35], a main effect of goal relevance [*F*(1,31) = 19.32, *p* < 0.01, $\eta_{p}^{2}$ = 0.38], and a two-way interaction that was marginally significant [*F*(1,31) = 4.11, *p* = 0.051, $\eta_{p}^{2}$ = 0.12]. Further simple-effects analyses showed that, when items were neutral, high goal relevance items were remembered better than low goal relevance items in the high salience condition [*F*(1,31) = 27.79, *p* < 0.01, $\eta_{p}^{2}$ = 0.47], but there was no difference between neutral high goal relevance items and neutral low goal relevance items in the low salience condition [*F*(1,31) = 3.31, *p* > 0.05]. Meanwhile, we also analyzed the effect of item salience and found in the high goal relevance condition, memory performance was greater for neutral high salience items compared to neutral low salience items [*F*(1,31) = 14.62, *p* < 0.01, $\eta_{p}^{2}$ = 0.32], while there was no difference between neutral high salience items and neutral low salience items in the low goal relevance condition [*F*(1,31) = 3.89, *p* > 0.05] (**Figure 2**).

Of note, there were also significant main effects of emotion type [*F*(1,31) = 52.58, *p* < 0.01, $\eta_{p}^{2}$ = 0.63], item salience [*F*(1,31) = 38.80, *p* < 0.01, $\eta_{p}^{2}$ = 0.56] and goal relevance [*F*(1,31) = 33.75, *p* < 0.01, $\eta_{p}^{2}$ = 0.52]. As expected, participants recognized more negative items (*M* = 0.61, *SD* = 0.01) than neutral items (*M* = 0.50, *SD* = 0.01), more high salience items (*M* = 0.61, *SD* = 0.01) than low salience items (*M* = 0.50, *SD* = 0.01), and more high relevance items (*M* = 0.63, *SD* = 0.02) than low relevance items (*M* = 0.48, *SD* = 0.01).

#### Item-Background Associative Memory

**FIGURE 3:**
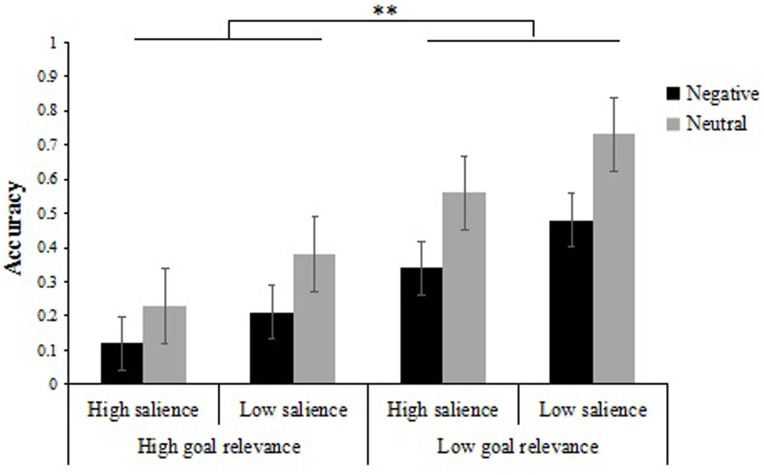
Recognition accuracy for intact associated backgrounds as a function of goal relevance, item salience and emotion type. Error bas represent standard error. ^∗∗^*p* < 0.01.

We also analyzed participants’ item-background associative memory performance (**Table 2**). A 2 (emotion type: negative, neutral) × 2 (item salience: low salience, high salience) × 2 (goal relevance: low relevance, high relevance) repeated measures ANOVA on item-background associative memory performance did not reveal a three-way interaction [*F*(1,31) = 0.21, *p >* 0.05]. Other results of interest related to interactions and main effects are as follows. There was a significant interaction between emotion type and goal relevance, *F*(1,31) = 6.05, *p* < 0.05, $\eta_{p}^{2}$ = 0.16. Further simple-effects analyses suggested that associative memory performance of low goal relevance items was greater than that of high goal relevance items regardless of the emotionality of items [*F*(1,31) = 70.27, *p* < 0.01, $\eta_{p}^{2}$ = 0.69; *F*(1,31) = 225.96, *p* < 0.01, $\eta_{p}^{2}$ = 0.88]. Meanwhile, we also analyzed the effect of emotion type and found associative memory performance was greater for neutral items compared with negative items in the high goal relevance condition and low goal relevance condition [*F*(1,31) = 37.93, *p* < 0.01, $\eta_{p}^{2}$ = 0.55; *F*(1,31) = 47.29, *p* < 0.01, $\eta_{p}^{2}$ = 0.60] (**Figure 3**). Besides, this ANOVA revealed the main effects of emotion type [*F*(1,31) = 83.85, *p* < 0.01, $\eta_{p}^{2}$ = 0.73], item salience [*F*(1,31) = 54.36, *p* < 0.01, $\eta_{p}^{2}$ = 0.64] and goal relevance [*F*(1,31) = 318.51, *p* < 0.01, $\eta_{p}^{2}$ = 0.91] were significant. More specifically, we found the ability for participants was greater to associate the backgrounds with neutral items (*M* = 0.47, *SD* = 0.01) than negative items (*M* = 0.29, *SD* = 0.01), with low salience items (*M* = 0.45, *SD* = 0.01) than high salience items (*M* = 0.31, *SD* = 0.01), and with low relevance items (*M* = 0.53, *SD* = 0.01) than high relevance items (*M* = 0.23, *SD* = 0.01).

**Table 2:** Accuracy for associative memory as a function of goal relevance, item salience, and emotion type (*M* ± *SD*).

	High goal relevance		Law goal relevance	
		
	High	Low	High	Low
	salience	salience	salience	salience
Negative	0.12 ± 0.13	0.21 ± 0.13	0.34 ± 0.15	0.48 ± 0.17
Neutral	0.23 ± 0.11	0.38 ± 0.11	0.56 ± 0.16	0.73 ± 0.18


Taken together, our outcomes are congruent with many findings, showing that emotion can improve the memory of the item itself, but this enhancement comes at the cost of impairing associative memory for emotional items and background information (Touryan et al., 2007; Mather and Knight, 2008; Mather et al., 2009). In addition, our results also indicated that such emotion effect was not simply due to a kind of attention capture elicited by emotion. Indeed, it also depended on the goal relevance and item salience of emotional items themselves. Specifically, we found high goal relevance and high item salience indeed enhanced emotional item memory performance while weakened the associative memory performance of emotional items and neutral backgrounds.

## Experiment 2

In Experiment 1, we manipulated perceptual feature of items by controlling their size and spatial location in background scenes. Furthermore, we wanted to know whether we could draw similar conclusions when perceptual feature of items was manipulated by controlling item familiarity. As mentioned above, item familiarity imparted through learning and subjective experience was different from objective perceptual feature via controlling size and location. Therefore, we further discussed the effects of emotion, goal relevance and item familiarity on associative memory in Experiment 2.

### Method

#### Participants

Thirty two undergraduate students from Shandong Normal University volunteered for the experiment (10 males and 22 females, mean age = 19.67 ± 2.50 years). None of the participants had a neurological or psychiatric history and all had normal or corrected-to-normal vision. All participants gave signed consent (approved by the local ethic committee) and received a gift after experiment.

#### Materials

One hundred and thirty six composite pictures with low perceptual-salience items were created and selected from materials in Experiment 1, 80 composite pictures were used as study materials while the remaining 56 composite pictures were used as new materials in recognition test phase. In addition, according to Funk and Hupbach’s (2014) study, we added 40 additional item images as filler items during item preexposure phase, which were not presented in the study and test phase.

#### Procedure

Participants first completed the item preexposure phase, in which they were presented with 80 item images in random order, including 40 neutral item images and 40 negative item images. Each item image was presented for 2 s followed by a 500-ms central fixation cross. Participants were asked to focus on these item images and to make efforts to remember them.

After the preexposure phase, participants were exposed to study phase and were presented with 40 negative composite pictures and 40 neutral composite pictures. Half of the negative and half of the neutral items contained in composite pictures had been preexposed. To ensure that all item-background composite pictures were presented for the same total duration in both the preexposure and the non-preexposure condition, composite pictures for which the item had been preexposed were presented for 3 s, composite pictures for which the item had not been preexposed were presented for 5 s. Apart from the different exposure time of item-background composite pictures, the procedure for study phase was same to that described in Experiment 1.

Following the study phase, participants needed to first complete a 3-min distractor task as Experiment 1 and then a memory test. At test, 56 old item images (28 preexposed items and 28 non-preexposed items) intermixed with 56 new item images were presented for old–new recognition. Following the participants’ response, we used a cued association memory test for each studied item, which was identical to that described in Experiment 1.

### Results and Discussion

#### Item Memory

**FIGURE 4:**
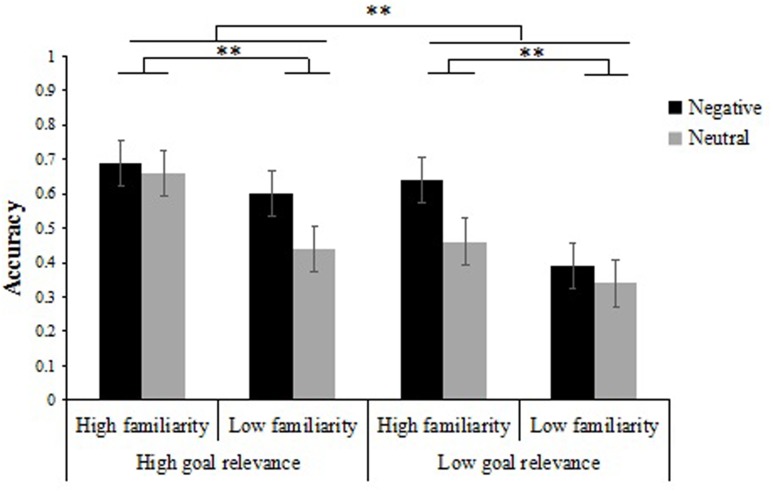
Recognition accuracy for items as a function of goal relevance, item familiarity and emotion type. Error bars represent standard error. ^∗∗^*p* < 0.01.

We analyzed the proportion of correct responses on the item memory test from Experiment 2 (**Table 3**) using a 2 × 2 × 2 repeated measures ANOVA with emotion type (negative vs. neutral), item familiarity (low familiarity vs. high familiarity) and goal relevance (low relevance vs. high relevance) as within-participant factors. Results showed a significant emotion type × goal relevance × item salience interaction [*F*(1,31) = 11.20, *p* < 0.01, $\eta_{p}^{2}$ = 0.27]. To further examine this interaction, we performed separate 2 × 2 ANOVAs on recognition of the items. Analysis of negative item memory performance was performed with item familiarity (low familiarity vs. high familiarity) and goal relevance (low relevance vs. high relevance) entered as within-participants factors. The results revealed a main effect of item familiarity [*F*(1,31) = 53.04, *p* < 0.01, $\eta_{p}^{2}$ = 0.63], a main effect of goal relevance [*F*(1,31) = 42.39, *p* < 0.01, $\eta_{p}^{2}$ = 0.58], and a significant interaction between item familiarity and goal relevance [*F*(1,31) = 12.88, *p* < 0.01, $\eta_{p}^{2}$ = 0.29]. Further simple-effects analyses showed that, when items were negative, memory performance for high goal relevance items was higher than that of low goal relevance items in the low familiarity condition [*F*(1,31) = 38.24, *p* < 0.01, $\eta_{p}^{2}$ = 0.55], whereas the memory performance did not differ between negative high goal relevance items and negative low goal relevance items in the high familiarity condition [*F*(1,31) = 3.75, *p* > 0.05]. Likewise, we also analyzed the effect of item familiarity and found no matter in the high goal relevance condition or in the low goal relevance condition, memory performance was greater for negative high familiarity items compared to negative low familiarity items [*F*(1,31) = 6.74, *p* < 0.05, $\eta_{p}^{2}$ = 0.18; *F*(1,31) = 69.05, *p* < 0.01, $\eta_{p}^{2}$ = 0.69] (**Figure 4**).

**Table 3:** Accuracy for item memory as a function of goal relevance, item familiarity, and emotion type (*M* ± *SD*).

	High goal relevance		Law goal relevance	
		
	High	Low	High	Low
	familiarity	familiarity	familiarity	familiarity
Negative	0.69 ± 0.13	0.60 ± 0.15	0.64 ± 0.10	0.39 ± 0.13
Neutral	0.66 ± 0.17	0.44 ± 0.17	0.46 ± 0.17	0.34 ± 0.19


Results from participants’ neutral item memory performance using a similar 2 × 2 ANOVA of item familiarity (low familiarity vs. high familiarity) and goal relevance (low relevance vs. high relevance) showed a main effect of item familiarity [*F*(1,31) = 26.97, *p* < 0.01, $\eta_{p}^{2}$ = 0.47], a main effect of goal relevance [*F*(1,31) = 27.07, *p* < 0.01, $\eta_{p}^{2}$ = 0.47], and a two-way interaction that was marginally significant [*F*(1,31) = 3.07, *p* = 0.08, $\eta_{p}^{2}$ = 0.09]. Further simple-effects analyses showed that, when items were neutral, memory performance for high goal relevance items was always greater than that of low goal relevance items no matter in the high familiarity condition or in the low familiarity condition [*F*(1,31) = 25.91, *p* < 0.01, $\eta_{p}^{2}$ = 0.46; *F*(1,31) = 4.58, *p* < 0.05, $\eta_{p}^{2}$ = 0.13]. Meanwhile, we also analyzed the effect of item familiarity and found, memory performance was greater for neutral high familiarity items compared to neutral low familiarity items in the high goal relevance condition and low goal relevance condition [*F*(1,31) = 25.26, *p* < 0.01, $\eta_{p}^{2}$ = 0.45; *F*(1,31) = 6.62, *p* < 0.05, $\eta_{p}^{2}$ = 0.18] (**Figure 4**).

In addition, analysis also revealed significant main effects of emotion type [*F*(1,31) = 27.90, *p* < 0.01, $\eta_{p}^{2}$ = 0.47], item familiarity [*F*(1,31) = 69.51, *p* < 0.01, $\eta_{p}^{2}$ = 0.69], and goal relevance [*F*(1,31) = 51.10, *p* < 0.01, $\eta_{p}^{2}$ = 0.62]. More specifically, participants recognized more negative items (*M* = 0.58, *SD* = 0.01) than neutral items (*M* = 0.48, *SD* = 0.02), more high familiarity items (*M* = 0.61, *SD* = 0.01) than low familiarity items (*M* = 0.44, *SD* = 0.02), and more high relevance items (*M* = 0.60, *SD* = 0.01) than low relevance items (*M* = 0.46, *SD* = 0.01).

#### Item-Background Associative Memory

**FIGURE 5:**
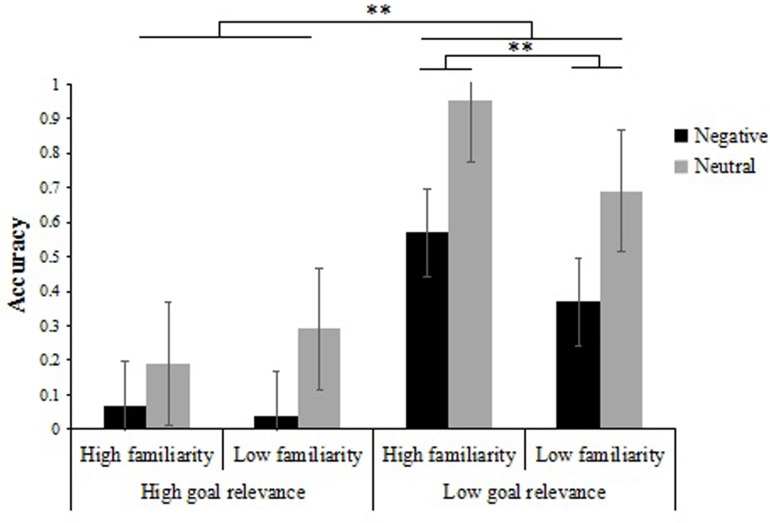
Recognition accuracy for intact associated backgrounds as a function of goal relevance, item familiarity, and emotion type. Error bas represent standard error. ^∗∗^*p* < 0.01.

Then, we continued to conduct a 2 × 2 × 2 repeated measures ANOVA on participants’ item-background associative memory scores (**Table 4**) with emotion type (negative vs. neutral), item familiarity (low familiarity vs. high familiarity) and goal relevance (low relevance vs. high relevance) as within-participant factors. The emotion type × item familiarity × goal relevance ANOVA revealed a significant three-way interaction, *F*(1,31) = 19.01, *p* < 0.01, $\eta_{p}^{2}$ = 0.38. To further analyze the interaction, we performed separate 2 × 2 ANOVAs on item-background associative memory. Analysis of participants’ associative memory performance for high familiarity items was performed with emotion type (negative vs. neutral) and goal relevance (low relevance vs. high relevance) entered as within-participants factors. The results revealed a main effect of emotion type [*F*(1,31) = 212.09, *p* < 0.01, $\eta_{p}^{2}$ = 0.87], a main effect of goal relevance [*F*(1,31) = 1912.11, *p* < 0.01, $\eta_{p}^{2}$ = 0.98], and a significant interaction between emotion type and goal relevance [*F*(1,31) = 72.48, *p* < 0.01, $\eta_{p}^{2}$ = 0.70]. Further simple-effects analyses showed that, when backgrounds associated with high familiarity items, associative memory performance for low goal relevance items was greater than that of high goal relevance items in negative and neutral conditions [*F*(1,31) = 442.86, *p* < 0.01, $\eta_{p}^{2}$ = 0.94; *F*(1,31) = 1899.04, *p* < 0.01, $\eta_{p}^{2}$ = 0.98] (**Figure 5**).

**Table 4:** Accuracy for associative memory as a function of goal relevance, item familiarity, and emotion type (*M* ± *SD*).

	High goal relevance		Law goal relevance	
		
	High	Low	High	Low
	familiarity	familiarity	familiarity	familiarity
Negative	0.07 ± 0.10	0.04 ± 0008	0.57 ± 0.11	0.37 ± 0.11
Neutral	0.19 ± 0.08	0.29 ± 0013	0.95 ± 0.09	0.69 ± 0.11


Results from participants’ associative memory performance for low familiarity items using a similar 2 × 2 ANOVA of emotion type (negative vs. neutral) and goal relevance (low relevance vs. high relevance) showed a main effect of emotion type [*F*(1,31) = 187.37, *p* < 0.01, $\eta_{p}^{2}$ = 0.86], a main effect of goal relevance [*F*(1,31) = 571.14, *p* < 0.01, $\eta_{p}^{2}$ = 0.95], and a marginally significant two-way interaction [*F*(1,31) = 3.33, *p* = 0.07, $\eta_{p}^{2}$ = 0.11]. Further simple-effects analyses showed that, when backgrounds associated with low familiarity items, associative memory performance for low goal relevance items was always greater than that of high goal relevance items no matter in negative condition or in neutral condition [*F*(1,31) = 242.56, *p* < 0.01, $\eta_{p}^{2}$ = 0.89; *F*(1,31) = 220.44, *p* < 0.01, $\eta_{p}^{2}$ = 0.88] (**Figure 5**).

Other results of interest related to interactions and main effects are as follows. There was a significant interaction between goal relevance and item familiarity, *F*(1,31) = 138.09, *p* < 0.01, $\eta_{p}^{2}$ = 0.82. Further examination of the interaction revealed that in the high goal relevance condition, there was no difference in associative memory performance between high familiarity and low familiarity items [*F*(1,31) = 2.48, *p* > 0.05], whereas in the low goal relevance condition, the associative memory performance of high familiarity items was better than that of low familiarity items [*F*(1,31) = 111.10, *p* < 0.01, $\eta_{p}^{2}$ = 0.78] (**Figure 5**). There was also a significant interaction between emotion type and goal relevance, *F*(1,31) = 37.69, *p* < 0.01, $\eta_{p}^{2}$ = 0.55. Further examination of the interaction indicated that associative memory performance of neutral items was better than that of negative items regardless of the goal relevance of items [*F*(1,31) = 103.83, *p* < 0.01, $\eta_{p}^{2}$ = 0.77; *F*(1,31) = 294.74, *p* < 0.01, $\eta_{p}^{2}$ = 0.91].

Additionally, the ANOVA revealed significant main effects of emotion type [*F*(1,31) = 349.69, *p* < 0.01, $\eta_{p}^{2}$ = 0.47], item familiarity [*F*(1,31) = 27.01, *p* < 0.01, $\eta_{p}^{2}$ = 0.27], and goal relevance [*F*(1,31) = 2586.50, *p* < 0.01, $\eta_{p}^{2}$ = 0.61]. Namely, the associative memory performance was greater for neutral items (*M* = 0.53, *SD* = 0.01) compared to negative items (*M* = 0.26, *SD* = 0.01), for high familiarity items (*M* = 0.45, *SD* = 0.01) compared to low familiarity items (*M* = 0.35, *SD* = 0.01), and for low relevance items (*M* = 0.65, *SD* = 0.01) compared to high relevance items (*M* = 0.15, *SD* = 0.01).

Consistent with Experiment 1, we also found both emotion and goal relevance had effects on item memory and associative memory. However, in contrast to Experiment 1, we found high familiarity could significantly improve the binding between emotional items and neutral backgrounds, which was consistent with the findings of Funk and Hupbach (2014). Additionally, the results from Experiment 2 showed a different effect of item familiarity on associative memory between high goal relevance condition and low goal relevance condition. Specifically, we found in the high goal relevance condition, there was no effect of familiarity; whereas in the low goal relevance condition, item familiarity could increase people’s ability to associate negative items and neutral backgrounds. These findings demonstrated the effect of item familiarity on associative memory would be modulated by goal relevance.

## General Discussion

In this study, we manipulated item priority during encoding by controlling both top–down goal relevance and bottom–up perceptual features. We investigated how goal relevance and perceptual features of emotional items influenced item-background associative memory in two experiments. Our results revealed that emotion could enhance item memory, but weaken associative memory. Goal relevance and perceptual features of emotional items jointly and interactively modulated the effect of emotion on associative memory.

Emotion could enhance item memory but impair memory for associated non-emotional background information. As a whole, our results showed that emotional items tend to be remembered better than neutral items, but memory for associated non-emotional background information tends to be impaired, which are congruent with many findings (Touryan et al., 2007; Mather and Knight, 2008; Mather et al., 2009). In other words, associations comprising emotional items and neutral backgrounds are more poorly remembered relative to those associations composed of neutral items and neutral backgrounds. Such findings could be explained by the attentional narrowing hypothesis (Easterbrook, 1959). Namely, emotion would narrow the focus of attention, capture more attention to emotional items but leave less attention to surrounding information, thus emotional items can be better remembered but surrounding information can be poorly remembered.

Item salience and item familiarity played different roles in emotional associative memory. In 2 experiments, goal relevance was manipulated in the same way by asking participants to pay attention to the foreground item images or the whole composite pictures, and perceptual feature was manipulated in different ways, by controlling the item salience in Experiment 1 and by controlling item familiarity in Experiment 2. The results from Experiment 1 showed that high salience could enhance emotional item memory but weaken the associative memory of emotional items and neutral backgrounds. However, the results of Experiment 2 showed high familiarity could enhance both emotional item memory and associative memory of emotional items and neutral backgrounds. We speculated the different outcomes in Experiment 1 and Experiment 2 may be related to the processing differences between item salience and item familiarity.

Although both item salience and item familiarity could be regarded as perceptual features, it was noteworthy that they still had some differences. Item salience reflected objective perceptual features of stimulus, while item familiarity reflected the effect of perceptual fluency, especially the familiarity induced by repeated presentation could improve the perceptual fluency (Begg et al., 1992; Westerman et al., 2002). Obviously, in Experiment 1, high salience of items (via visual contrast) could automatically capture more attention resources and thus suppressed processing of the background scene. Similar results also have been observed in the study by Lee et al. (2012). In contrast, in Experiment 2, high familiarity of items could enhance perceptual fluency of items, thus people might need less attentional or cognitive resources for item encoding and could pay more attention to background scenes. In this way, the associations between items and backgrounds would be promoted.

Goal relevance and perceptual features of items jointly and interactively modulated the effect of emotion on item-background associative memory. The results from Experiment 1 showed that both goal relevance and perceptual features of items jointly modulated the effect of emotion on item-background associative memory, and high goal relevance did weaken the associative memory of emotional items and neutral backgrounds. Indeed, we considered that in the high goal relevance condition, participants were just asked to focus on item images, so it was no doubt that items grabbing more attention would be remembered better than unnoticed backgrounds. Also, Sakaki et al. (2013) showed that emotion could strengthen the memory for stimuli relevant to current goals, but weaken the memory for stimuli irrelevant to goals. In Experiment 2, although high familiarity and high goal relevance could enhance item memory of emotional items, they had interactive effects on associative memory of emotional items and neutral backgrounds. Specifically, the effect of item familiarity would be suppressed in the high goal relevance condition; whereas in the low goal relevance condition, high familiarity could increase people’s ability to associate negative items and neutral backgrounds. Such findings reflected the role of item familiarity on associative memory for emotional items would be affected by goal relevance.

What might account for the different outcomes between Experiment 1 and Experiment 2? We speculated that it might be related to different perceptual features between item salience and item familiarity. High salient items could automatically capture more attention resources, and thus less likely be influenced by goal relevance. However, though high familiarity could enhance perceptual fluency of items, high familiarity could not automatically capture attention on items. Meanwhile, high goal relevance also required people to divide attention to items. Therefore, when people needed to process items consciously in both high familiarity and high goal relevance conditions, undoubtedly that the effect of item familiarity on associative memory for emotional items would be modulated by goal relevance. Indeed, prioritizing goal relevant information and ignoring irrelevant stimuli is vital in our daily life, especially under emotion condition. People must learn the most important aspects of emotional events without being distracted by other details to increase survival in danger.

Taken together, we could emphasize the important roles of goal relevance and perceptual features of emotional items on associative memory. That is, although emotion might weaken memory for associated non-emotional background information, the impairment of emotion on associative memory for background information could be limited to some degree and could be modulated by top–down goal relevance and bottom–up perceptual features. Especially, high familiarity of items could promote associative memory between emotional items and neutral backgrounds. Therefore, on the basis of these findings, our study indicated that the effects of emotion on associative memory was not only related to attention capture elicited by emotion, but also depended on goal relevance and perceptual features of emotional items.

It was noteworthy that in this study, we just manipulated goal relevance and perceptual features of emotional items in the encoding phase by using visual materials. So further studies would pay more attention to the effects of goal relevance and perceptual features of emotional items on associative memory from the perspective of retrieving phase, and widen the experiment materials by using more real world auditory or video manipulations.

## Ethics Statement

Ethical approval for this study was obtained from the Academic Board of Shandong Normal University. This manuscript has not been submitted elsewhere for publication, in whole or in part and will not be submitted elsewhere during the review process. All participants provided written informed consent and all procedures used in studies involving human committee of Shandong Normal University. This institutional academic committee is responsible for ethical review.

## Author Contributions

WM is in charge of instruction of design and explanation of whole experiments, paper writing and modification; SA is in charge of paper writing and XY is in charge of experiment explanation and data collection.

## Conflict of Interest Statement

The authors declare that the research was conducted in the absence of any commercial or financial relationships that could be construed as a potential conflict of interest.
